# Clinical Efficacy of Empirical Therapy in Children with Vasovagal Syncope

**DOI:** 10.3390/children9071065

**Published:** 2022-07-17

**Authors:** Chunyan Tao, Yaxi Cui, Chunyu Zhang, Xueqin Liu, Qingyou Zhang, Ping Liu, Yuli Wang, Junbao Du, Hongfang Jin

**Affiliations:** 1 Department of Pediatrics, Peking University First Hospital, Beijing 100034, China; taocy126@126.com (C.T.); tracycuiyaxi@gmail.com (Y.C.); chunyu5781@163.com (C.Z.); 101811liu@163.com (X.L.); zhangqingyou_73@126.com (Q.Z.); duoduomama19731013@126.com (P.L.); mengyu1129@126.com (Y.W.); junbaodu1@126.com (J.D.); 2 Department of Genetics and Endocrinology, Guangzhou Women and Children’s Medical Center, Guangzhou Medical University, Guangzhou 510623, China; 3 Key Laboratory of Molecular Cardiovascular Sciences, Ministry of Education, Beijing 100191, China

**Keywords:** children, efficacy, empiric therapy, vasovagal syncope

## Abstract

(1) Background: This case-control study was designed to assess the efficacy of empiric treatment for vasovagal syncope in children; (2) Methods: We retrospectively enrolled 181 children with vasovagal syncope from the Department of Pediatrics of Peking University First Hospital. The participants were categorized into four groups, based on the empiric treatment received: conventional treatment, including health education and orthostatic training; conventional treatment plus oral rehydration salts; conventional treatment plus metoprolol; conventional treatment plus midodrine hydrochloride. Patients were followed up to evaluate the syncopal or presyncopal recurrence. Kaplan–Meier curves were drawn to explore the syncopal or presyncopal recurrence in children, and the differences were compared among the groups using a log-rank test; (3) Results: Among the 181 children with vasovagal syncope, 11 were lost to follow-up. The median time of follow-up was 20 (8, 42) months. The Kaplan–Meier survival curve showed no significant difference in syncopal or presyncopal recurrence in children treated with different empiric options according to a log-rank test (*χ^2^* = 1.328, *p* = 0.723); (4) Conclusions: The efficacy of unselected empiric therapy of vasovagal syncope in children was limited, and the individualized therapies merit further studies.

## 1. Introduction

Syncope is a common emergency condition caused by a temporary interruption of cerebral perfusion, characterized by a transient loss of consciousness and muscle tone, followed by fainting [1,2]. Syncope in pediatric patients can be subdivided into neuro-mediated syncope, cardiogenic syncope, and cerebrovascular syncope, among which vasovagal syncope, the main type of neurally mediated syncope, accounts for 30–80% of all syncope cases [3,4,5]. A survey showed that 15% of children aged <18 years experienced syncope at least once [6]. Statistically, 16–49% of children with syncope will suffer unintentional injuries resulting from syncope episodes [7,8]. In addition, recurrent syncope or anxiety about recurrent syncope can prevent some patients from attending school and participating in daily activities, which significantly reduces their quality of life and seriously affects their physical and mental health [9,10,11]. The children significantly affected by syncope need timely treatment to improve their quality of life.

Due to the limited knowledge of pediatric vasovagal syncope, clinicians generally make therapeutic decisions based on their own experience. Conventional therapy, including health education and orthostatic training, is commonly applied. In addition, oral rehydration salts, β-blockers, or α-agonists are empirically used in some of the institutions [12]. However, the pathogenesis of vasovagal syncope is complex, and clinical experience suggests that it is difficult to achieve a satisfactory therapeutic effect using only non-targeted therapies [13,14]. Therefore, the present study aimed to analyze the clinical therapeutic effect of untargeted empiric therapies on children with vasovagal syncope.

## 2. Materials and Methods

### 2.1. Study Subjects

This is a retrospective case-control study. A total of 181 children with vasovagal syncope from the Department of Pediatrics, Peking University First Hospital of China were enrolled. The exclusion criteria included liver dysfunction, renal dysfunction, myocarditis, nervous system diseases, and psychological diseases. The participants, 108 girls (60%) and 73 boys (40%), aged 10.9 ± 2.7 years, were categorized into four groups, based on the empirical treatment received: (1) conventional treatment involving health education and orthostatic training; (2) conventional treatment plus oral rehydration salts; (3) conventional treatment plus metoprolol; and (4) conventional treatment plus midodrine hydrochloride. The medical records of the cases were obtained from the electronic medical record system (Kaihua, Beijing, China).

The diagnostic criteria for vasovagal syncope in children are as follows [12,15,16]: (1) patients with syncope or presyncope; (2) patients who occasionally experience triggers before syncopal episodes, such as standing for a long time, muggy environment, exposure to emotional stress, pain, or medical settings; (3) patients with a positive head-up tilt test; and (4) exclusion of cardiovascular organic diseases, cerebrovascular diseases, metabolic diseases, and cardiogenic diseases.

The study was conducted in line with the Helsinki Declaration and was approved by the Ethics Committee of Peking University First Hospital in China (2018 [202]). All of the study participants or their parents signed an informed consent form.

### 2.2. Head-Up Tilt Test

The head-up tilt test was performed in a warm, quiet, dimly lit environment. The participants were required to stop taking any medications that would affect autonomic nervous activity for at least five half-life periods, and to fast for at least 4 h. After the bladder was emptied, the child was required to lie on the tilt bed (SHUT-100A, Standard, China) for 10 min; the bed was then tilted at 60 degrees for 45 min. A Finapres Medical System-FMS (FinometerPRO, FMS, Amsterdam, Netherlands) was used for continuously monitoring the heart rate (HR), blood pressure (BP), and electrocardiogram data of the patients. The test would be terminated quickly when children had positive responses, otherwise, the tilt position would be maintained for 45 min [17]. The positive response criteria were syncope or presyncopal symptoms, such as headache, dizziness, heart palpitations, chest tightness, nausea, abdominal pain, and sweating, accompanied by one of the following: (1) BP was decreased significantly (systolic BP of children ˂ 80 mmHg or diastolic BP ˂ 50 mmHg, or a >25% decrease in mean BP); (2) HR slowed (HR of children aged 4–6 years < 75 bpm; HR of children aged 7–8 years < 65 bpm; HR of children aged > 8 years < 60 bpm); (3) Electrocardiogram showed sinus arrest for over 3 s or was replaced by a junctional escape rhythm; (4) atrioventricular conduction block of second-degree or greater [18,19]. Vasovagal syncope was further classified as vasodepressor, cardioinhibitory, or mixed type, based on the type of hemodynamic changes in the head-up tilt test.

### 2.3. Treatment and Follow-Up Protocol

**Figure 1:**
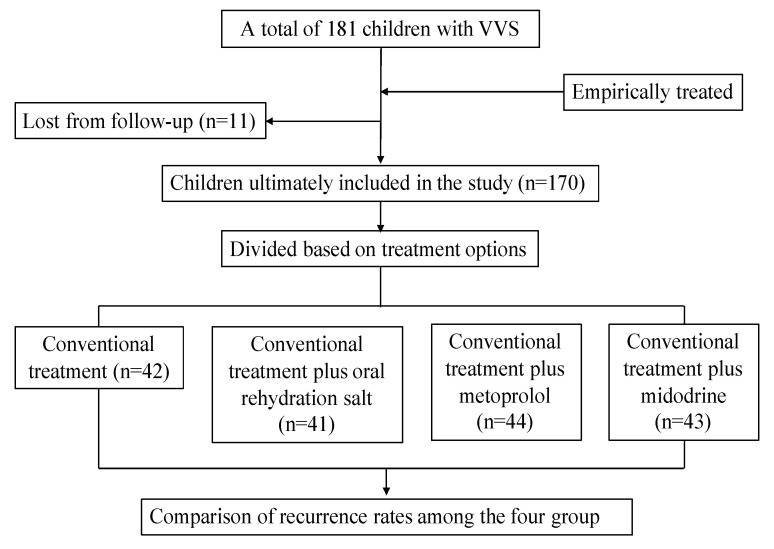
Flowchart of children with vasovagal syncope. VVS: vasovagal syncope.

The patients who had only one episode of syncope usually received conventional treatment, and the other patients who had more than one episode of syncope usually empirically received conventional treatment plus oral rehydration salts, conventional treatment plus metoprolol, or conventional treatment plus midodrine hydrochloride (Figure 1). The conventional treatment consisted of health education, avoiding triggers that cause fainting, drinking plenty of water, and orthostatic training. The oral rehydration salts were prescribed at 5.125 g/day and were dissolved in 250–500 mL water. Metoprolol (AstraZeneca, London, UK) was prescribed at 0.5 mg·kg^−1^/day twice a day. Midodrine (Sinopharm Chuankang, Chengdu, China) was prescribed at 1.25–5 mg/day once a day.

The children were followed up by a professional pediatrician at a pediatric cardiology clinic or by telephone. The start of the follow-up was set as the beginning of treatment, and the mean follow-up time of cases was 20 (8, 42) months. During the follow-up, the recurrence of syncopal or presyncopal episodes was evaluated. The recurrence rate was determined by dividing the number of children who had episodes of syncopal or presyncopal occurrence during the follow-up period by the number of cases in the group.

### 2.4. Statistical Process and Analysis

The analysis was performed with SPSS 25.0 software (IBM, New York, NY, USA). The data normality was tested with the Shapiro–Wilk test. Normally distributed continuous data are presented as mean ± standard deviation, and non-normally distributed continuous data are presented as median and interquartile range. The difference in the normally distributed data between the two groups was compared with an independent *t*-test. The difference in the non-normally distributed data between the two groups was compared with the Mann–Whitney *U*-test. The ANOVA was used to compare the data among the groups and LSD was used for multiple post-comparison in the normally distributed data. The Kruskal–Wallis test was used to compare non-normally distributed data among the groups. The categorical data were expressed as frequency (percentage). The Chi-square analysis was used to compare the categorical data among groups. The survival curves were drawn with the Kaplan–Meier method, and the log-rank test was performed to compare the differences among the different groups. *p* < 0.05 was regarded as statistically significant.

## 3. Results

### 3.1. Characteristics of Participants

Among the 181 participants suffering from vasovagal syncope who received different therapies, 11 (6%) were lost to follow-up. There were no differences in demographic characteristics (sex and visiting age), medical history characteristics (age of onset, course, frequency of syncope or presyncope, length of syncope episode, syncope-related injury, allergy history, and family history of syncope), and the types of vasovagal syncope between the 11 cases lost to follow-up and the other cases retained for analysis (*p* > 0.05, Table 1).

**Figure 2:**
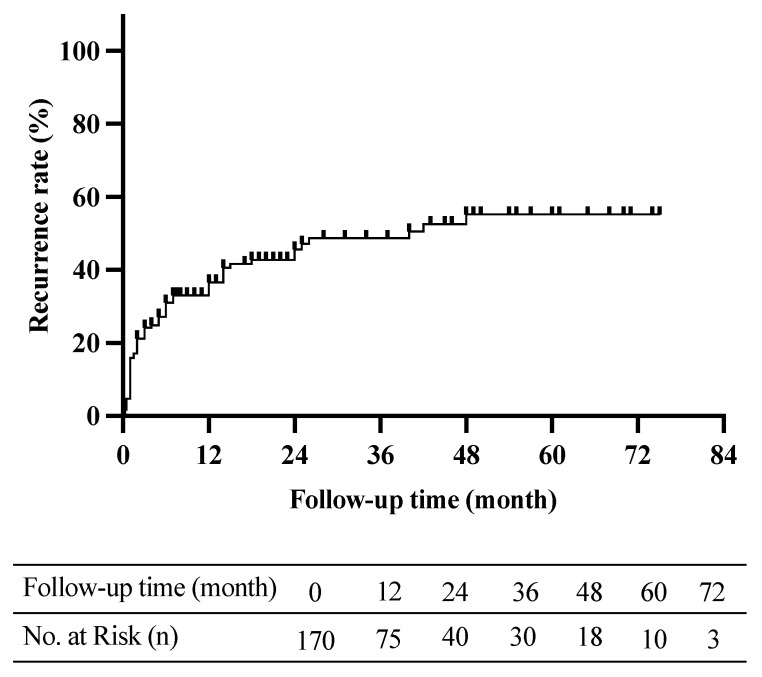
Kaplan–Meier curve analysis of children with vasovagal syncope during follow-up (*n* = 170). The mean follow-up time was 20 (8, 42) months, and 72 children (42%) experienced syncopal or presyncopal recurrence.

The average age of the children was 10.9 ± 2.7 years, and the age at the initial episode of syncope or presyncope was 8.8 ± 3.3 years. The mean course of the follow-up cases before admission was 12.0 (3.0, 36.0) months. The participants experienced an average of 4.0 (2.0, 6.0) episodes of syncope or presyncope in the follow-up period. The median syncope or presyncope frequencies were 0.25 (0.12, 1.00) syncopal or presyncopal episodes per month before treatment in the conventional treatment group, 0.26 (0.13, 1.00), in the conventional treatment plus oral rehydration salts group, 0.33 (0.11, 1.65) in the conventional treatment plus metoprolol group, and 0.33 (0.10, 1.00) in the conventional treatment plus midodrine hydrochloride group. There was no obvious difference in the baseline frequencies of syncope among the four treatment groups (*p* > 0.05). We followed up the 170 participants (102 girls and 68 boys) for a mean of 20 (8, 42) months. Overall, 72 children (42%) experienced syncopal or presyncopal recurrence (Figure 2). Among them, 42 patients (58%) experienced their first recurrence before the end of treatment, while the remaining 30 patients (42%) suffered their first recurrence after the end of treatment.

The vasodepressor type accounted for 72% (122/170) of the cases, the mixed type accounted for 26% (44/170), and the cardioinhibitory type accounted for 2% (four cases). The mean time for a positive response to occur during the head-up tilt test was 18 (9, 31) min.

### 3.2. Comparison of Baseline Characteristics among Study Participants Receiving Different Treatment Options

Among the 170 follow-up participants, 42 received conventional treatment, 41 received conventional treatment plus oral rehydration salts, 44 received conventional treatment plus metoprolol, and 43 received conventional treatment plus midodrine hydrochloride. There were no marked differences in the age, sex, course, age of onset, frequency of syncope or presyncope, lasting time of syncope, syncope-related injury, allergy history, family history of syncope, and types of vasovagal syncope in the children among the four groups (*p* > 0.05, Table 2).

### 3.3. Comparison of Syncopal or Presyncopal Recurrence among Participants Receiving Different Treatment Options

**Figure 3:**
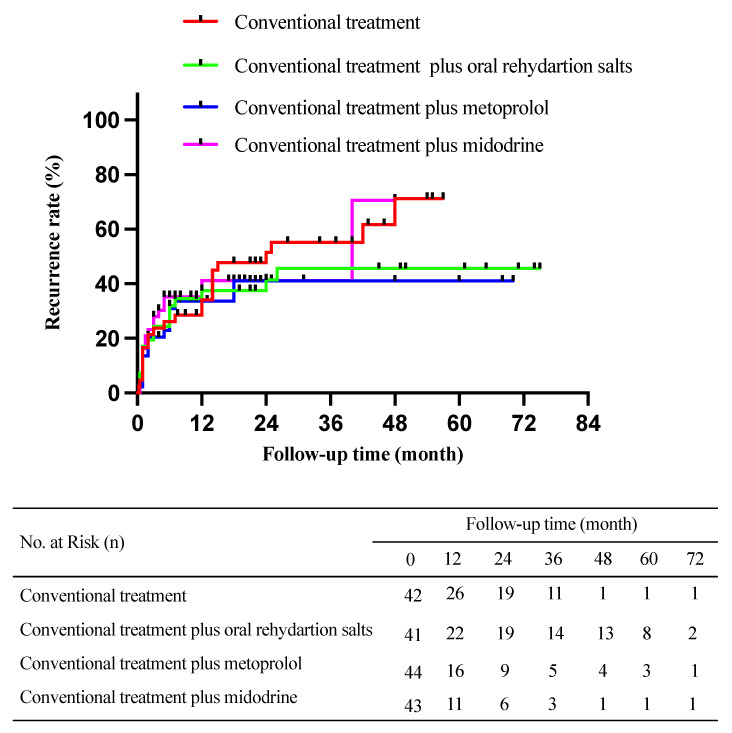
Kaplan–Meier curve analysis with the log-rank test for the recurrence of vasovagal syncope in different treatment groups. The mean follow-up time was 20 (8, 42) months. Overall, 23/42 (55%) cases in the conventional treatment group, 17/41 (41%) cases in the conventional treatment plus oral rehydration salts group, 15/44 (34%) cases in the conventional treatment plus metoprolol group, and 17/43 (40%) cases in the conventional treatment plus midodrine hydrochloride group had a syncopal or presyncopal recurrence.***χ^2^*** = 1.328, *p* = 0.723.

The recurrence rate of syncope or presyncope was 55% for the patients who received conventional treatment, 41% for those who received conventional treatment plus oral rehydration salts, 34% for those who received conventional treatment plus metoprolol, and 40% for those who received conventional treatment plus midodrine hydrochloride. The log-rank test showed no obvious difference in the syncopal or presyncopal recurrence rate among the four groups (*χ^2^* = 1.328, *p* = 0.723, Figure 3).

## 4. Discussion

In this study, 42% of the children with vasovagal syncope experienced syncopal or presyncopal recurrence during a long-term follow-up. No significant differences in syncopal or presyncopal recurrence were found among the participants receiving the different treatment modalities during follow-up.

Previous studies have shown discrepant findings in the efficacy of the treatment for vasovagal syncope. The efficacy of several different therapeutic strategies proposed in the guidelines has been controversial [20]. Some studies indicated that tilt training, oral rehydration salts, or metoprolol showed effectiveness in some patients with vasovagal syncope [21,22,23,24]. One clinical trial previously demonstrated that midodrine hydrochloride was effective for preventing syncope and reducing the recurrence of syncope [13,25]. However, On et al. observed that the recurrence of syncope and presyncope was 42.9% in the tilt-training group and 47.1% in the control group, over a 16.9 ± 3.1 month-follow-up (*p* = 0.815) [26]. Bellard et al. found that the positive rate on the head-up tilt test did not decrease in the patients suffering from vasovagal syncope who received an increased salt and fluid intake, compared to that in the patients who did not receive treatment, suggesting that the treatment was ineffective [27]. Zhang et al. showed that there was no marked difference in the recurrence rates of syncope between the metoprolol group (43%) and the control group (29%) during a 22 ± 10 month-follow-up (*p* = 0.389) [28]. Romme et al. found that the syncopal and presyncopal recurrence did not differ significantly between the midodrine hydrochloride and placebo treatment groups in patients who failed to respond to non-pharmacologic treatment (*p* > 0.05), in a randomized cross-over trial [29]. The above data suggest that the efficacy of the empirical unselected use of any treatment is limited and discrepant.

In the present study, we first followed up the pediatric patients who received the four empirical modalities in the treatment of vasovagal syncope in children, including conventional treatment, conventional treatment plus oral rehydration salts, conventional treatment plus metoprolol, and conventional treatment plus midodrine hydrochloride. We observed that nearly half of the children reported a recurrence of syncope or presyncope during approximately 2 years of follow-up, and that the recurrence rates did not statistically differ among the four treatment modalities. The results suggested that the effectiveness of empirical unselected conventional or different empirical pharmacological modalities for pediatric vasovagal syncope was limited if the treatment was implemented, regardless of the mechanisms underlying vasovagal syncope.

Therefore, individualized therapy, instead of empirical unselected therapy, might be a promising treatment strategy. The limited therapeutic values indicated that different mechanisms might require different treatments. The mechanisms underlying vasovagal syncope have not been clarified [30]; however, the main mechanisms include autonomic dysfunction or excessive baseline catecholamine status, hypovolemia, and peripheral vascular dysfunction [31]. Orthostatic training is expected to be useful for vasovagal syncope in children with autonomic dysfunction as the main mechanism [32]. The increased fluid intake can increase the circulating blood volume, enhance the stability of the autonomic nervous system, and increase vascular resistance [33]. Metoprolol targets the adrenaline receptor and blocks the function of the increased catecholamines, and slows down the HR [34]. Metoprolol treatment is considered to be effective for those patients with excessive baseline catecholamine status as the main underlying mechanism [35]. Midodrine hydrochloride is a representative agent of α-receptor agonists, which can constrict blood vessels and increase vascular resistance [36]; it is expected to be effective for vasovagal syncope cases with peripheral vascular dysfunction [25]. Therefore, it seems that orthostatic training, oral rehydration salts, metoprolol, and midodrine hydrochloride function via different mechanisms. The continuous development of biological technologies and the progress of evidence-based medicine will promote the development of individualized therapies [37].

This study also has several limitations that should be noted. This is a single-center and small sample-sized retrospective study, with recalling bias and confounding variables. In the present study, we followed up and compared the four empirical treatment modalities of vasovagal syncope in children for the first time, and suggested that the effectiveness of empirical pharmacologic modalities for pediatric vasovagal syncope was limited. To improve the therapeutic efficacy, future research investigating the efficacy of individualized treatment targeting the different main mechanisms underlying vasovagal syncope in children is required.

## Figures and Tables

**Table 1:** Clinical characteristics of study participants who completed and lost to follow-up.

**Follow-Up**	***n***	**Sex (Female %)**	**Visiting Age (Year)**	**Age at Onset (Year)**	**Course of Illness (Month) ^a^**	**Episodes of Syncope and Presyncope ^a^**
Completed	170	102 (60)	10.9 ± 2.7	8.8 ± 3.3	12.0 (3.0, 36.0)	4.0 (2.0, 6.0)
Lost	11	6 (55)	11.9 ± 3.9	10.1 ± 4.6	14.0 (4.0, 36.0)	3.0 (2.0, 12.0)
Z/*χ^2^*	-	0.002	−1.328	−1.225	−0.518	−0.749
*p* value	-	>0.05	>0.05	>0.05	>0.05	>0.05
**Follow-Up**	***n***	**Time of TLOC ≥ 1 min (%)**	**Syncope-Related Injury (%)**	**Allergic History (%)**	**Positive Family History (%)**	**Type of Syncope (Vasodepressor Type %)**
Completed	170	112 (66)	19 (11)	45 (26)	41 (24)	122 (72)
Lost	11	7 (64)	0 (0)	4 (36)	2 (18)	7 (64)
*Z/* *χ* ^2^	-	0.023	1.374	0.512	0.201	0.055
*p* value	-	>0.05	>0.05	>0.05	>0.05	>0.05

TLOC: Transient loss of consciousness. ^a^ non-normal distribution data compared using the Mann–Whitney *U*-test.

**Table 2:** Analysis of clinical characteristics among the different treatment groups.

**Group**	***n***	**Sex (Female %)**	**Visiting Age (Year)**	**Age at Onset (Year)**	**Course of Illness (Month) ^b^**	**Episodes of Syncope and Presyncope ^b^**
Conventional treatment	42	26 (62)	10.3 ± 2.5	8.6 ± 3.1	12.0 (1.8, 30.0)	3.0 (2.0, 6.0)
Conventional treatment plus oral rehydration salts	41	21 (51)	10.5 ± 2.7 ^a^	8.7 ± 2.9	12.0 (2.5, 35.0)	4.0 (2.5, 6.0)
Conventional treatment plus metoprolol	44	30 (68)	11.6 ± 2.5	9.4 ± 3.7 ^a^	11.5 (3.6, 45.0)	4.0 (3.0, 7.0)
Conventional treatment plus midodrine hydrochloride	43	25 (58)	11.0 ± 2.8 ^a^	8.6 ± 3.2 ^a^	13.0 (4.0, 48.0)	5.0 (3.0, 7.0)
H/*χ*^2^	-	2.670	2.380	0.527	0.893	5.307
*p* value	-	>0.05	>0.05	>0.05	>0.05	>0.05
**Group**	***n***	**Time of TLOC ≥ 1 min (%)**	**Syncope-Related Injury (%)**	**Allergic History (%)**	**Positive Family History (%)**	**Type of Syncope (Vasodepressor Type %)**
Conventional treatment	42	25 (60)	4 (10)	11 (26)	11 (26)	28 (67)
Conventional treatment plus oral rehydration salts	41	28 (68)	3 (7)	11 (27)	15 (37)	28 (68)
Conventional treatment plus metoprolol	44	30 (68)	6 (14)	14 (32)	8 (18)	34 (77)
Conventional treatment plus midodrine hydrochloride	43	29 (67)	6 (14)	9 (21)	7 (16)	32 (74)
*χ^2^*	-	1.011	1.333	1.329	5.872	1.591
*p* value	-	>0.05	>0.05	>0.05	>0.05	>0.05

TLOC: Transient loss of consciousness. ^a^ non-normal distribution data compared using the Mann–Whitney *U*-test. ^b^ non-normal distribution data compared using the Kruskal–Wallis test.

## Data Availability

The data are available from the corresponding author on reasonable request.
